# HIV and Hepatitis C Virus Testing Delays at Methadone Clinics in Guangdong Province, China

**DOI:** 10.1371/journal.pone.0066787

**Published:** 2013-06-20

**Authors:** Ying-Hua Xia, Megan M. McLaughlin, Wen Chen, Li Ling, Joseph D. Tucker

**Affiliations:** 1 Faculty of Medical Statistics and Epidemiology, School of Public Health, Sun Yat-sen University, Guangzhou, P.R. China; 2 Sun Yat-sen Center for Migrant Health Policy, Sun Yat-sen University, Guangzhou, P.R. China; 3 UNC Project-China, Guangzhou, P.R. China; British Columbia Centre for Excellence in HIV/AIDS, Canada

## Abstract

In China, injection drug use is a major transmission route for HIV and hepatitis C virus (HCV) infection. Timely HIV and HCV testing among drug users is vital to earlier diagnosis, linkage to care, and retention. This study aimed to examine HIV and hepatitis C virus (HCV) testing delays at methadone clinics in Guangdong Province, China, and identify individual-level and clinic-level factors associated with delayed testing. Data from 13,270 individuals at 45 methadone clinics in Guangdong were abstracted from a national web-based surveillance database. A two-level binomial logit model was used to examine the association between individual- and clinic-level factors and delayed HIV and HCV testing, defined as receiving a test seven or more days after initial entry into the methadone system. Among 10,046 patients tested for HIV, 1882 (18.7%) had delayed testing; among 10,404 patients tested for HCV, 1542 (14.8%) had delayed testing. Among delayed testers, the median time to HCV testing was significantly longer than the median time to HIV testing (73 vs. 54 days, p<0.05). In the multivariate analysis, the likelihood of delayed HIV testing was higher among individuals with high school or greater education (adjusted odds ratio [aOR] 1.32, 95% confidence interval [CI] 1.02–1.72) and individuals enrolled at clinics with more patients (aOR 1.41, 95% CI 1.05–1.91, for each increase in 100). The likelihood of delayed HCV testing was higher among women (aOR 1.51, 95% CI 1.11–2.06) and employed individuals (aOR 1.21, 95% CI 1.02–1.43). Delayed testing for HIV and HCV is common among patients at methadone clinics in Guangdong, with many patients experiencing delays of two or more months. Structural interventions are needed to expedite testing once individuals enter the methadone maintenance program.

## Introduction

Timely HIV testing among most-at-risk populations is vital to earlier diagnosis, linkage to care, and retention. Although HIV testing is currently accessible in a variety of health care settings in many countries, delayed diagnosis continues to be a common problem worldwide [1], [2], [3], [4], [5]. The clinical and public health benefits of expedited HIV treatment were demonstrated in the HIV Prevention Trials Network (HPTN) 052 trial, which showed a large reduction in HIV transmission and death or serious opportunistic infections in the early therapy group [6]. In contrast, diagnostic and therapeutic delay in HIV-infected patients can lead to increased risk of ongoing transmission [7], morbidity [8], and mortality [9].

In China, late HIV diagnosis continues to play a substantial role in late access to ART [10], [11]. Early diagnosis and enrollment in care is particularly important in a setting like China, where a large proportion of patients diagnosed HIV-positive are already considerably immunosuppressed [10]. For these patients, earlier identification could have a large impact in terms of opportunistic infections and care required, with implications for the patients’ long-term morbidity and mortality. Similarly, earlier detection of hepatitis C virus (HCV) infection can allow for earlier initiation of therapy, which in turn can prolong survival and prevent cirrhosis and hepatocellular carcinoma [12].

In China, where injection drug use is a major transmission route for HIV and HCV infection affects nearly half of injection drug users [13], national guidelines recommend routine opt-out, provider-initiated HIV and HCV testing for all patients at methadone clinics upon entry. Injection drug users face numerous barriers to accessing health care services, including stigmatization and criminalization of drug use. Methadone clinics offer a unique opportunity to access this hard-to-reach population and provide care. Approximately 700 methadone clinics serve 300,000 drug users nationwide [14]. However, retention rates in methadone maintenance programs in China are low [14], [15], [16], with as many as a quarter of the patients dropping out within one month of enrollment [16]. Thus, expediting HIV and HCV testing is important for ensuring that infected patients complete CD4 cell count testing, disease staging, and initiation of treatment while they are actively enrolled in methadone maintenance services.

Individual-level factors, such as younger age and lower educational status, that might influence patients’ initial willingness to undergo testing can lead to delays in HIV and HCV testing. Additionally, clinic-level factors–staffing, intake protocols, and quality of pre-test counseling–can contribute to testing delays. Most studies of expedited HIV testing have focused on improving individual test-seeking behavior [3], [5], neglecting the institutional and structural factors that also contribute to delayed HIV testing. We examine HIV and HCV testing delays at methadone clinics in Guangdong Province, China and identify individual-level and clinic-level factors associated with delayed testing.

## Methods

### Ethics Statement

This study was approved by the Health Bureau of Guangdong Province and the Ethics Review Committee at Sun Yat-sen University. Written informed consent was provided by clinical directors who answered the questionnaire. All patients gave their written consent for their information to be stored in the national web-based administrative database and allowed to use for research.

### National Web-based Disease Surveillance Database of the Methadone Maintenance Program

A national unified web-based HIV/AIDS information system has been established by the Chinese Centers for Disease Control and Prevention (CDC). Details of this system have been described elsewhere [17]. Methadone maintenance treatment is one of its eight core subsystems, and the only subsystem conducting opt-out, provider-initiated routine testing among drug users. Clinic clients include individuals who self-refer and those who are enrolled as part of a post-incarceration rehabilitation plan.

According to national guidelines, providers at methadone clinics routinely conduct HIV and HCV testing when clients register at methadone clinics and enter results into the web-based management database [18]. In addition, individual socio-demographic characteristics, drug use behavior, and daily methadone dosage are also entered into the database.

### HIV and HCV Treatment

Individuals who test positive for HIV or HCV at methadone clinics are referred by clinic staff to outside health care facilities for further evaluation and treatment. HIV- and HCV-infected patients are treated according to national guidelines [19], [20]. HIV patients undergo clinical and immunological staging, and those with CD4 cell counts ≤350×10^6^/L or AIDS-defining illnesses are initiated on standardized first-line treatment regimens that typically consist of three antiretroviral drugs [19]. HCV patients are classified as having chronic or acute HCV infection based on clinical and laboratory examinations and initiated on therapy if indicated [20].

### Setting

Of the people living with HIV in China who were infected through injecting drug use, 84.2% live in six of the country’s 33 provinces/autonomous regions, including Guangdong Province [21]. A systematic review estimated that the prevalence of HIV and HCV among drug users in Guangdong was 2.44% and 44.6% respectively [13]. At the time of this study, 49 methadone maintenance clinics were established in Guangdong, covering 18 of the 23 cities in the province. This cross-sectional study was conducted in all methadone clinics in Guangdong.

### Measures

Our primary outcome was delayed testing, a dichotomous outcome defined as receiving an HIV or HCV test seven or more days after initial entry into the methadone system, which was based on the date the blood sample was collected. This cutoff was chosen because according to national guidelines seven days is the threshold for considering someone a drop-out from the methadone maintenance program [18]. Additionally, high rates of early attrition from the methadone program have been observed in Guangdong, with 24.1% of enrolled patients dropping out by the end of the first month [16]. Early testing is necessary to ensure that these high-risk patients are tested before they become lost to follow-up. Thus, we used a strict definition for timely HIV and HCV testing in methadone clinics in China.

Individual-level socio-demographic and clinical characteristics included in the analysis were sex, age (<29 years old, 30–39 years old, >40 years old), marital status (married, unmarried), education completed (none/primary school, middle school, high school and above), employment status (employed, unemployed), and drug injecting experience (injecting, non-injecting), HIV infection status (infected, non-infected), HCV infection status (infected, non-infected), and years of heroin use (≤5, 5 to 10, >10). Clinic-level factors included were economic region (Pearl River Delta, non-Pearl River Delta), administrative level (town/village level, city level), affiliated institution (CDC, non-CDC), number of nurses and physicians employed (2–4, 5–7, 8–10), number of clients (<200, 200–399, ≥400), integration of voluntary HIV counseling services (yes, no), clinic finances (for-profit, non-profit), and distance between the clinic and most clients’ residences (≤5 kilometers, >5 kilometers).

### Data Collection

Data were collected during November and December 2008. All individual characteristics were obtained from the web-based management system. Clinical staff downloaded the data and removed all identifying information. Clinic characteristics were obtained through a self-administered questionnaire completed by the director of each methadone clinic.

### Data Analysis

Four clinics were opened less than one month prior to the study and were not included in the analysis. A total of 13,442 individuals were registered in the remaining 45 methadone clinics in Guangdong Province at the time of study. 172 (1.28%) individuals were excluded from the analysis because they had three or more individual-level variables missing from their dataset.

First, we calculated the percentage of patients who had not yet been tested for HIV and HCV at 3 days, 5 days, 7 days, 15 days, and 1 month, among individuals who eventually underwent testing, and examined the time trend. Second, we examined the univariate associations between individual-level and clinic-level characteristics and delayed HIV and HCV testing. Group comparisons were examined with chi-square tests at the individual level and with nonparametric Kruskal-Wallis tests at the clinic level.

We observed clustering of observations at the clinic level and decided to use multilevel modeling in order to examine the interactions between individual- and clinic-level factors. A two-level binomial logit model was used to estimate the effect of individual- and clinic-level factors on delayed HIV and HCV testing. The form and interpretation of the model are described elsewhere [22]. In a sensitivity analysis, we re-estimated the multilevel model using 5 days and 10 days as the cutoff for delayed testing. The multilevel analysis was conducted using MLwiN Version 2.02 (Center for Multilevel Modelling, University of Bristol, Bristol, UK), and all other analyses were conducted using SAS Version 9.1 (SAS Institute Inc, Cary, NC, USA). Reported p-values were two-sided, and a p-value below 0.05 was considered significant.

## Results

Data from 13,270 individuals at 45 clinics were analyzed. The majority of individuals were male (93.9%) and heroin users (99.6%), and 82.6% were between 20 and 40 years old. Most individuals had completed nine years or less of school and were unemployed at the time of the survey (Table 1).

**Table 1:** Individual-level characteristics of individuals at methadone clinics in Guangdong Province (N = 13,270).

Characteristic	N (%) total individuals	N (%) individuals tested for HIV	N (%) individuals tested for HCV
Overall	13270 (100)	10046 (100)	10404 (100)
Sex			
Male	12463 (93.9)	9400 (93.6)	9753 (93.7)
Female	807 (6.1)	646 (6.4)	651 (6.3)
Age (years)			
<20	36 (0.3)	25 (0.2)	25 (0.2)
20–29	3242 (24.4)	2346 (23.4)	2411 (23.2)
30–39	7725 (58.2)	5907 (58.5)	6148 (59.1)
40–49	2080 (15.7)	1621 (16.1)	1664 (16.0)
50–59	177 (1.3)	140 (1.4)	149 (1.4)
>59	10 (0.1)	7 (0.1)	7 (0.1)
Marital status			
Married	6446 (48.6)	4911 (48.9)	5079 (48.8)
Unmarried	6824 (51.4)	5135 (51.1)	5325 (51.2)
Education			
None/primary school	2760 (20.8)	1695 (16.9)	2158 (20.7)
Middle school	8325 (62.7)	6292 (62.6)	6517 (62.6)
High school and above	2185 (16.5)	2059 (20.5)	1729 (16.6)
Employment status			
Employed	4926 (37.1)	3752 (37.3)	3650 (35.1)
Unemployed	8344 (62.9)	6294 (62.7)	6754 (64.9)
Ever injected drugs			
Yes	11011 (83.0)	8348 (83.1)	8663 (83.3)
No	2259 (17.0)	1698 (16.9)	1741 (16.7)
Years of heroin use			
≤5	2642 (19.9)	1876 (18.7)	1910 (18.4)
5 to 10	3578 (27.0)	2694 (26.8)	2794 (26.8)
>10	7050 (53.1)	5476 (54.5)	5700 (54.8)

Among the 13,270 individuals analyzed, 10,046 (75.7%) had an HIV test and 10,404 (78.4%) underwent HCV testing. Among those who ultimately were tested, 1882 (18.7%) had delayed HIV testing and 1542 (14.8%) had delayed HCV testing. Among delayed testers, the median time to HIV testing was 54 days (range, 8–731) and the median time to HCV testing was 73 days (range, 8–997). More individuals had delayed HIV test uptake compared to HCV test uptake (p<0.05, see Figure S1). After one month, 77.4% and 66.1% of the delayed HIV and HCV testers, respectively, had still not received testing.

**Figure 1:**
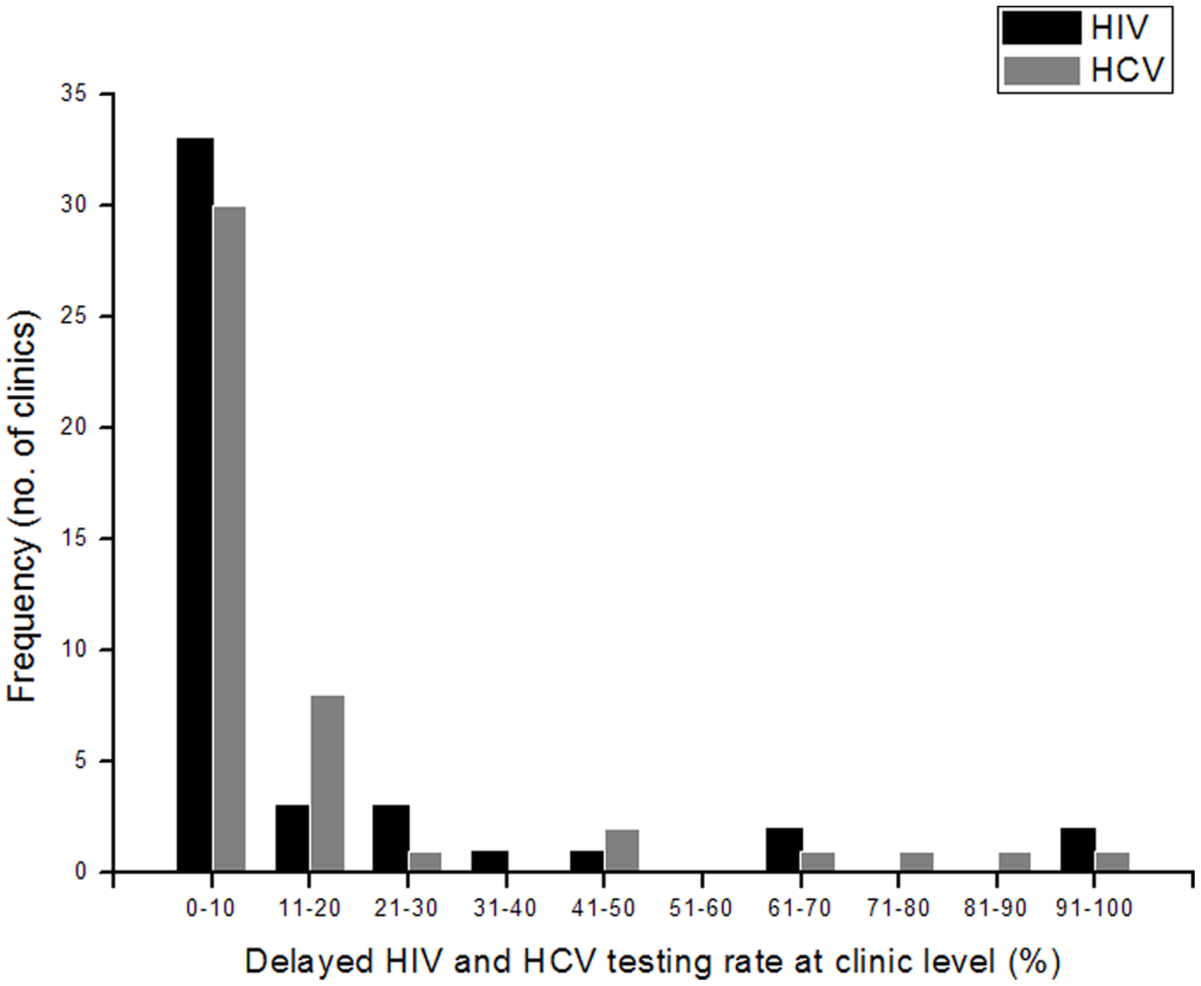
Distribution of delayed HIV and HCV testing rates at methadone clinics in Guangdong, China.

The majority of methadone clinics had HIV and HCV testing delays that affected less than 10% of the clients who ultimately were tested (Figure 1). At a small number of clinics, however, more than 40% of clients tested experienced HIV or HCV testing delays. In most cases, the clinics with a high percentage of delayed HIV testing were not the same ones with a high percentage of delayed HCV testing.

In the univariate analysis, several individual-level factors were associated with delayed HIV testing, but no clinic-level factors were found to be significant (see Table S1). These individual-level factors were younger age, unmarried, high school level or above education, non-injecting drug use, HIV-negative serostatus, and years of heroin use. The final two-level model predicting delayed HIV testing included one individual-level factor (higher education) and one clinic-level factor (larger number of clients enrolled) (Table 2). Compared with individuals who received no education or only finished primary school, individuals who finished high school or above were more likely to have delayed HIV testing (adjusted odds ratio [aOR] 1.32, 95% confidence interval [CI] 1.02–1.72). Enrollment at methadone clinics with a larger number of patients was also associated with increased likelihood of delayed HIV testing (aOR 1.41, 95% CI 1.05–1.91).

**Table 2:** Multivariate analysis of factors associated with delayed HIV testing at methadone clinics (N = 10,046).

Characteristic	N (%) patients with delayed HIV testing	Adjusted odds ratio (95%confidence interval)	p-value
Overall	1882 (18.7)	NA	
Sex			
Male	1750 (18.6)	–	–
Female	132 (20.4)	–	–
Age			
≤29 years old	526 (22.2)	–	–
30–39 years old	1081 (18.3)	–	–
≥40 years old	275 (15.6)	–	–
Marital status			
Married	884 (17.2)	–	–
Unmarried	998 (20.3)	–	–
Education			
None/primary school	382 (18.6)	1.00	
Middle school	1147 (18.2)	1.17 (0.96–1.44)	0.122
High school and above	353 (20.8)	1.32 (1.02–1.72)	0.036
Employment status			
Unemployed	1200 (19.1)	–	–
Employed	682 (18.2)	–	–
Ever injected drugs			
Yes	1474 (17.7)	–	–
No	408 (24.0)	–	–
HIV			
Infected	64 (10.7)	–	–
Non-infected	1818 (19.2)	–	–
HCV*			
Infected	1219 (19.4)	–	–
Non-infected	560 (19.3)	–	–
Years of heroin use			
≤5	501 (26.7)	–	–
5 to 10	561 (20.8)	–	–
>10	820 (15.0)	–	–
Total number of clients (for each increase in 100) **	-	1.41 (1.05–1.91)	0.024

* 103 individuals who had delayed HIV testing did not undergo HCV testing.

** Total number of clients is the only clinic-level variable associated with delayed HIV testing in the multivariate analysis. Other non-significant clinic-level variables are not presented in the table. The complete results of the univariate analysis of clinic-level variables are presented in Table S1.

The factors associated with delayed HCV testing differed from those associated with delayed HIV testing. Significant factors in the univariate analysis included younger age, employment, and non-injecting drug use. The final two-level model predicting delayed HCV testing included sex and employment status (Table 3). Women were more likely to have delayed HCV compared to men (aOR 1.51, 95% CI 1.11–2.06), and employed individuals were more likely than unemployed individuals to have delayed HCV testing rate (aOR 1.21, 95% CI 1.02–1.43). No clinic-level factor was significantly associated delayed HCV testing in the multivariate analysis.

**Table 3:** Multivariate analysis of factors associated with delayed HCV testing at methadone clinics (N = 10,404).

Characteristic	N (%) patients withdelayed HCV testing	Adjusted odds ratio (95% confidence interval)	p-value
Overall	1542 (14.8)	NA	
Sex			
Male	1437 (14.7)	1.00	
Female	105 (16.1)	1.51 (1.11–2.06)	0.013
Age			
≤29 years old	403 (16.5)	–	–
30–39 years old	922 (15.0)	–	–
≥40 years old	217 (11.9)	–	–
Marital status			
Married	764 (14.4)	–	–
Unmarried	778 (15.3)	–	–
Education			
None/primary school	308 (14.3)	–	–
Middle school	985 (15.1)	–	–
High school and above	249 (14.4)	–	–
Employment status			
Unemployed	933 (13.8)	1.00	
Employed	609 (16.7)	1.21 (1.02–1.43)	0.032
Ever injected drugs			
Yes	1236 (14.3)	–	–
No	306 (17.6)	–	–
HIV*			
Infected	75 (16.3)	–	–
Non-infected	1271 (14.6)	–	–
HCV			
Infected	1061 (14.9)	–	–
Non-infected	481 (14.6)	–	–
Years of heroin use			
≤5	289 (15.1)	–	–
5 to 10	449 (16.1)	–	–
>10	804 (14.1)	–	–

* 196 individuals who had delayed HCV testing did not undergo HIV testing.

Note: Only individual-level variables are included in this table. None of the clinical-level variables were associated with delayed HCV testing in the final multilevel model. The complete results of the univariate analysis of clinic-level variables are presented in Table S1.

In the sensitivity analysis, the multivariate model for delayed HIV testing did not change when the threshold for delayed testing was increased from 7 to 10 days. However, education dropped out of the multivariate model when the cutoff was reduced to 5 days. In all three multivariate models, the relationship between clinic volume and delayed HIV testing remained significant (see Table S2). For HCV, changing the threshold to 5 days resulted in gender dropping out of the multivariate model. When the cutoff was increased to 10 days, the significance of the gender and employment variables was reduced but the p-value remained less than 0.1 (see Table S3).

## Discussion

Effective systems of HIV and HCV care require prompt testing, linkage to care, and retention. This study in Guangdong Province, China revealed that delays in HIV and HCV testing are common at methadone clinics and often persist for months. A total of 18.7% and 14.8% of HIV and HCV testers, respectively, were delayed in receiving tests, despite being enrolled in the methadone clinic system. Among delayed testers, the median delay to testing was 54 days for HIV and 73 days for HCV.

Although the importance of preventing delayed HIV and HCV testing has been recognized, studies of testing delays often focus on individual patient characteristics associated with late testing and neglect the important role of institutional and systems issues. Structural factors associated with delays in time to testing once individual patients have accessed facilities offering HIV testing have been understudied and underappreciated. Additionally, delays in HCV testing among high-risk groups like injection drug users have been understudied compared to delayed HIV diagnosis. Methadone clinics provide a unique opportunity to explore how to optimize these systems in order to expedite HIV and HCV testing for drug users. To our knowledge, this is the first study of structural factors associated with delayed HIV and HCV testing in methadone clinics.

Existing definitions of delayed testing used in previous studies have a number of limitations. Studies of HIV testing delays commonly examine the outcome of delayed diagnosis, which is defined according to CD4 count at the time of diagnosis [1], [2], [3], [4], [23]. For HCV, delayed diagnosis has been defined as the time from the estimated date of infection (date of initiation of injection drug use) to the diagnosis date [24]. These definitions do not distinguish between patient delays in accessing sites where testing is available and delays that occur once patients have presented for care at these sites. Other studies have reported the occurrence of “missed opportunities,” defined as documented clinical encounters when patients with clinical syndromes associated with HIV or patients belonging to high-risk groups did not undergo testing [25], [26], [27]. Two previous studies have reported the median time to HIV testing following patients’ initial encounter with the health facility [25], [28]. However, there is no established threshold for measuring delayed testing. Moreover, these studies were undertaken among outpatients at general hospitals and clinics, not in methadone clinic settings where patients are evaluated on a daily basis.

Given the limitations of the existing literature, we used an operational definition of seven or more days from enrollment to testing to define delayed testing. We based our definition on the number of days since enrollment because we sought to examine the institutional issues that affect time to testing after patients’ enrollment in the methadone maintenance program. We chose our cutoff for delayed testing with the understanding that methadone clinics differ from other health care settings in two key ways–the patients are evaluated on a daily basis, and they are at high-risk for HIV and HCV infection. Given the high rates of early attrition documented at methadone clinics in Guangdong [16], we used a strict definition for timely testing (<7 days) in methadone clinics in China. The testing delays documented in this study demonstrate that there is room for improvement within the medical system to achieve timely HIV and HCV testing among drug users. More research is needed to better understand how to decrease time to testing within medical infrastructures.

We found that the median delay to HCV testing was longer than the median delay to HIV testing by almost three weeks. Previous studies in Canada and Europe have identified poor perception of the risk of HCV infection and the harm caused by HCV as the main reason for delayed or missed HCV testing or failure to seek treatment [29], [30]. Inadequate education about HCV among drug users in China may result in poor recognition of the importance of HCV testing and longer testing delays compared to HIV [31]. Perceptions of lower HCV risk among women may also account for relationship between female gender and delayed HCV testing found in our study.

Our study found that methadone clinics seeing a greater number of patients had more frequent HIV test delay. Methadone clinic staff shortage is a national problem in China and may partly explain this relationship [32]. Health care providers in a typical methadone clinic in China experience a heavy workload, a problem that has also been reported in the U.S [33]. Often two or three clinicians are managing care for more than 100 patients, including writing prescriptions, providing counseling and health education, tracking patients lost to follow-up, and preparing reports [32]. The relationship between number of clients and delayed testing was not observed for HCV. We hypothesize that this may be because HIV counseling may require more staff time in order to be effective; since HIV is both more stigmatized and less prevalent compared to HCV, more intensive counseling may be needed to overcome individuals’ resistance to testing at entry to the methadone maintenance program.

A limitation of this study is that the methadone clinics in Guangdong Province may differ from others in China in terms of the type of testing technology used and existing relationships with local hospitals to expedite routine screening. However, there were no HIV or HCV point-of-care tests available in China at the time of this study. Moreover, the characteristics of the study population were comparable with those reported at methadone clinics in other parts of China [15]. Another limitation is that we cannot evaluate the clinical impact of delayed HIV or HCV testing. Individuals who test positive HIV and HCV at methadone clinics are referred to outside health care facilities for further evaluation and HIV and HCV treatment. Their treatment data, including timing of treatment initiation and CD4 counts, are recorded in a separate administrative system maintained by the local CDC, which was not accessible for the current study. Finally, patients in the methadone program not only differ in the characteristics we examined in this study, but also in their dependence level and mental health. We did not include these factors because the methadone maintenance program system does not routinely collect information about mental health and dependence level, but these factors may affect time to testing.

A strength of this study is that we included data from nearly all of the 49 methadone clinics in Guangdong Province. Only four clinics that opened less than one month prior to the study were not included in the analysis. Another major strength of our study is that it examines salient clinic-level factors that could be modified to improve routine, prompt HIV and HCV testing in methadone clinics. Our results suggest that methadone clinics that serve a large number of patients may require capacity strengthening in order to manage their patient load and prevent HIV testing delays. Other clinic-level variables that we examined were not significantly associated with testing delays in the multivariate model. However, examination of the percentage of delayed testing at the clinic level revealed that there is a small subset of methadone clinics where the percentage of clients who experience delayed HIV or HCV testing is higher than average. Clinic-level variables that we did not examine–such as measures of staff buy-in, staff training, or clinic resources–may account for this clinic-level variation. Further research is needed to understand why these clinics perform poorly on timeliness of HIV and HCV testing.

Although current national guidelines recommend routine opt-out, provider-initiated HIV and HCV testing for all patients at methadone clinics upon entry, testing procedures may need to be further integrated into the intake protocols of methadone clinics in order to ensure implementation of prompt testing. For example, the intake protocol could require that providers offer HIV and HCV testing routinely at multiple specified times during the first week after entry to the methadone maintenance program. Such redundancy early in the process could help to ensure that initial patient refusal or individual providers’ failure to offer testing at intake not result in prolonged testing delays.

Expediting HIV and HCV testing among drug users in China is particularly important because they are a high-risk and hard-to-reach population [34]. Methadone clinics are a key point of contact between drug users and the Chinese health care system. The cascade of services that must be completed for optimal care of HIV or HCV infection includes initial screening, testing confirmation, linkage to care, disease staging, initiation of treatment, and retention in care [35]. The existing infrastructure of methadone clinics could be used to enhance care for drug users infected with HIV or HCV if these services could be further streamlined. Methadone clinics in China face a high rate of early attrition [16]. Thus, prompt HIV and HCV testing after intake is important for ensuring patients complete the full cascade of HIV and HCV care services while still enrolled in the methadone maintenance program.

## Supporting Information

Figure S1 Percentage of delayed HIV and HCV testing by the number of days after enrollment at the methadone clinic.
(TIF)

Table S1 Clinic-level characteristics associated with delayed HIV and HCV testing at methadone clinics in Guangdong Province (N = 45).
(DOC)

Table S2 Multivariate analysis of factors associated with delayed HIV testing at methadone clinics (N = 10,046).
(DOC)

Table S3 Multivariate analysis of factors associated with delayed HCV testing at methadone clinics (N = 10,404).
(DOC)
