# Supplementary figures and images for: HIV and Hepatitis C Virus Testing Delays at Methadone Clinics in Guangdong Province, China
Source: PLoS One. 2013 Jun 20;8(6):e66787. doi: 10.1371/journal.pone.0066787 (PMC3688574; doi:10.1371/journal.pone.0066787)

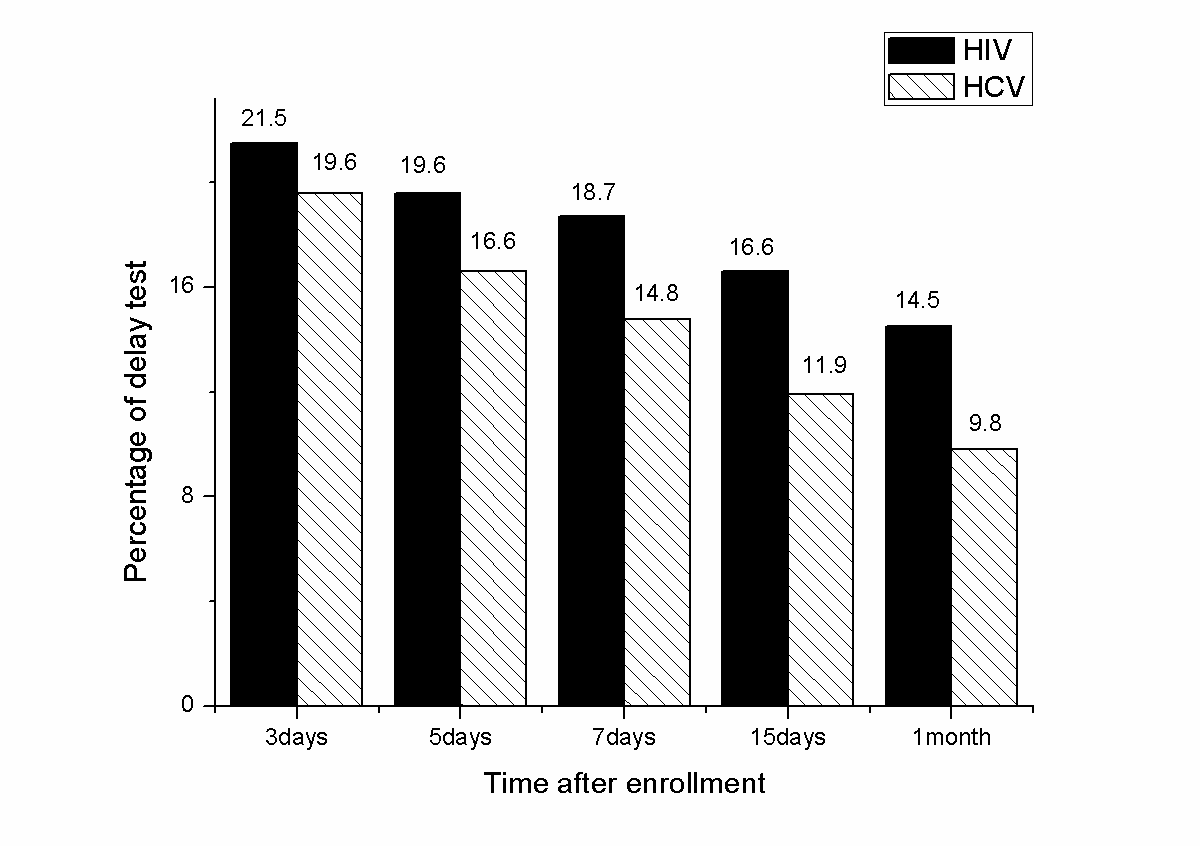

Supplement: Figure S1 — Percentage of delayed HIV and HCV testing by the number of days after enrollment at the methadone clinic. (TIF) [file pone.0066787.s001.tif]
