# Supplementary material for: HIV and Hepatitis C Virus Testing Delays at Methadone Clinics in Guangdong Province, China
Source: PLoS One. 2013 Jun 20;8(6):e66787. doi: 10.1371/journal.pone.0066787 (PMC3688574; doi:10.1371/journal.pone.0066787)
Supplement: Table S1 — Clinic-level characteristics associated with delayed HIV and HCV testing at methadone clinics in Guangdong Province (N = 45). (DOC) [file pone.0066787.s002.doc]

**TABLE S1. Clinic-level characteristics associated with delayed HIV and HCV testing at methadone clinics in Guangdong Province (N=45)**

| Variables | N (%) clinics | Delayed HIV testing (%) | | | Delayed HCV testing (%) | | |
| --- | --- | --- | --- | --- | --- | --- | --- |
|  |  | Median | Interquartile range | p-value | Median | Interquartile range | p-value |
| Economic region |  |  |  |  |  |  |  |
| Pearl River Delta* | 25 (55.6) | 3.57 | 0.84-8.33 | 0.273 | 4.46 | 1.21-13.75 | 0.840 |
| Non-Pearl River Delta | 20 (44.4) | 5.52 | 2.15-27.93 |  | 3.43 | 2.50-18.34 |  |
| Administrative level |  |  |  |  |  |  |  |
| Town/village level | 8 (17.8) | 2.77 | 1.20-4.56 | 0.130 | 2.69 | 1.02-9.34 | 0.315 |
| City level | 37 (82.2) | 5.77 | 1.64-15.35 |  | 4.40 | 2.61-17.31 |  |
| Affiliated institution** |  |  |  |  |  |  |  |
| Center for Disease Control and Prevention (CDC) | 13 (28.9) | 3.59 | 0.74-27.14 | 0.920 | 5.77 | 2.65-16.28 | 0.787 |
| Non-CDC | 32 (71.1) | 4.47 | 1.79-9.98 |  | 3.57 | 1.21-15.25 |  |
| Number of healthcare personnel |  |  |  |  |  |  |  |
| 2-4 | 13(28.9) | 6.08 | 2.75-15.35 | 0.189 | 6.28 | 2.84-17.14 | 0.649 |
| 5-7 | 27(60.0) | 2.67 | 0.59-7.94 |  | 3.23 | 0.83-16.28 |  |
| 8-10 | 5(11.1) | 11.39 | 3.57-11.48 |  | 3.57 | 3.48-13.75 |  |
| Total number of clients |  |  |  |  |  |  |  |
| <200 | 17(37.8) | 3.57 | 1.94-8.57 | 0.767 | 3.23 | 2.38-13.75 | 0.437 |
| 200-399 | 17(37.8) | 3.59 | 1.64-11.48 |  | 4.35 | 2.80-16.28 |  |
| 400 | 11(24.4) | 6.92 | 0.74-68.65 |  | 7.22 | 1.21-67.98 |  |
| Integration of voluntary HIV counseling and testing |  |  |  |  |  |  |  |
| Yes | 29(64.4) | 4.72 | 1.64-11.48 | 0.537 | 3.48 | 1.21-19.03 | 0.806 |
| No | 16(35.6) | 3.12 | 1.39-9.98 |  | 4.10 | 2.65-15.25 |  |
| Distance from most clients’ residence to clinic |  |  |  |  |  |  |  |
| ≤5km | 20(44.4) | 4.99 | 1.73-8.13 | 0.982 | 4.35 | 3.11-15.25 | 0.514 |
| >5km | 25(55.6) | 2.74 | 1.64-15.35 |  | 2.88 | 0.83-18.34 |  |
| For-profit clinic |  |  |  |  |  |  |  |
| Yes | 19(42.2) | 4.90 | 0.82-27.14 |  | 6.28 | 2.84-19.03 |  |
| No | 26(57.8) | 3.30 | 1.94-8.33 |  | 3.40 | 0.83-11.31 |  |

*Pearl River Delta: A dense network of economically developed cities in South China

**Affiliated institution: A methadone clinic is not an independently operated institution. Clinics are affiliated with institutions such as a local Center for Disease Control and Prevention, public comprehensive hospital, or specialist hospital. The affiliated institution manages the clinic, is responsible for administrative issues, and provides human resources and equipment to the clinic.
