# Supplementary material for: HIV and Hepatitis C Virus Testing Delays at Methadone Clinics in Guangdong Province, China
Source: PLoS One. 2013 Jun 20;8(6):e66787. doi: 10.1371/journal.pone.0066787 (PMC3688574; doi:10.1371/journal.pone.0066787)
Supplement: Table S2 — Multivariate analysis of factors associated with delayed HIV testing at methadone clinics (N = 10,046). (DOC) [file pone.0066787.s003.doc]

**TABLE S2. Multivariate analysis of factors associated with delayed HIV testing at methadone clinics (N=10,046)**

|  |  | 5 days |  |  | 7 days |  |  | 10 days |  |
| --- | --- | --- | --- | --- | --- | --- | --- | --- | --- |
| Characteristic | N (%) patients with delayed HIV testing | Adjusted odds ratio (95% confidence interval) | p-value | N (%) patients with delayed HIV testing | Adjusted odds ratio (95% confidence interval) | p-value | N (%) patients with delayed HIV testing | Adjusted odds ratio (95% confidence interval) | p-value |
| N | 1968 (19.6) |  |  | 1882 (18.7) |  |  | 1781 (17.7) |  |  |
| Education |  |  |  |  |  |  |  |  |  |
| None/primary school | 409 (19.9) | — | — | 382 (18.6) | 1.00 |  | 382 (18.6) | 1.00 |  |
| Middle school | 1194 (19.0) | — | — | 1147 (18.2) | 1.17 (0.96-1.44) | 0.122 | 1147 (18.2) | 1.16 (0.94-1.43) | 0.178 |
| High school and above | 365 (21.5) | — | — | 353 (20.8) | 1.32 (1.02-1.72) | 0.036 | 353 (20.8) | 1.32 (1.00-1.73) | 0.047 |
| Total number of clients (for each increase in 100) ** | - | 1.32 (1.04-1.67) | 0.023 | - | 1.41 (1.05-1.91) | 0.024 | - | 1.48 (1.07-2.03) | 0.018 |
