# Supplementary material for: HIV and Hepatitis C Virus Testing Delays at Methadone Clinics in Guangdong Province, China
Source: PLoS One. 2013 Jun 20;8(6):e66787. doi: 10.1371/journal.pone.0066787 (PMC3688574; doi:10.1371/journal.pone.0066787)
Supplement: Table S3 — Multivariate analysis of factors associated with delayed HCV testing at methadone clinics (N = 10,404). (DOC) [file pone.0066787.s004.doc]

**TABLE S3. Multivariate analysis of factors associated with delayed HCV testing at methadone clinics (N=10,404)**

|  |  | 5 days |  |  | 7 days |  |  | 10 days |  |
| --- | --- | --- | --- | --- | --- | --- | --- | --- | --- |
| Characteristic | N (%) patients with delayed HCV testing | Adjusted odds ratio (95% confidence interval) | p-value | N (%) patients with delayed HCV testing | Adjusted odds ratio (95% confidence interval) | p-value | N (%) patients with delayed HCV testing | Adjusted odds ratio (95% confidence interval) | p-value |
| N | 1730 (16.6) |  |  | 1542 (14.8) |  |  | 1398 (13.4) |  |  |
| Sex |  |  |  |  |  |  |  |  |  |
| Male | 1616 (16.6) | — | — | 1437 (14.7) | 1.00 |  | 1307 (13.4) | 1.00 |  |
| Female | 114 (17.5) | — | — | 105 (16.1) | 1.51 (1.11-2.06) | 0.013 | 91 (14.0) | 1.36 (0.97-1.92) | 0.07 |
| Employment status |  |  |  |  |  |  |  |  |  |
| Unemployed | 1071 (15.9) | 1.00 |  | 933 (13.8) | 1.00 |  | 829 (12.3) | 1.00 |  |
| Employed | 659 (18.1) | 1.13 (1.00-1.27) | 0.039 | 609 (16.7) | 1.21 (1.02-1.43) | 0.032 | 569 (15.6) | 1.17 (0.98-1.41) | 0.09 |
